# A network-based brain stimulation paradigm for alcohol use disorder with co-occurring major depressive disorder: a mini review targeting the ventral attention network

**DOI:** 10.3389/fneur.2026.1810254

**Published:** 2026-07-23

**Authors:** Marc L. Copersino, Matthew S. Gage, Emma Joncas, Agustin G. Yip, Rujuta Ulhas Parlikar, Mark A. Halko

**Affiliations:** 1 McLean Hospital, Belmont, MA, United States; 2 Department of Psychiatry, Harvard Medical School, Boston, MA, United States

**Keywords:** alcohol use disorder, attention networks, major depressive disorder, network-based neuromodulation, salience network, TMS, transcranial magnetic stimulation, ventral attention network

## Abstract

Alcohol use disorder (AUD) is frequently complicated by co-occurring major depressive disorder (MDD), a comorbidity associated with greater clinical severity, elevated relapse risk, and poorer treatment outcomes than either condition alone. Despite the prevalence and burden of this dual diagnosis, interventions that simultaneously address alcohol use and depressive symptoms show limited effectiveness. Converging evidence from neuroimaging and translational research indicates that AUD and MDD share disruptions across large-scale brain networks involved in salience processing, attentional control, and affect regulation. In this Mini Review, we synthesize the literature on transcranial magnetic stimulation (TMS) in AUD, highlighting dominant region-centric paradigms (including those adapted from major depressive disorder), methodological heterogeneity, and ongoing uncertainties regarding optimal targets and mechanisms of action. We then outline a network-based framework centered on the ventral attention network (VAN) as an alternative neuromodulation approach for AUD with co-occurring MDD. Drawing on attention neuroscience and systems-level models of addiction, we emphasize the temporoparietal junction as a theoretically grounded and neuromodulation-accessible entry point for probing VAN responsivity and plasticity. By reframing TMS as a mechanistic probe of network dynamics rather than solely as a therapeutic intervention, this perspective provides a foundation for developing more precise, biologically grounded approaches to addressing heterogeneity and relapse vulnerability in this clinically complex population.

## Introduction

Alcohol use disorder (AUD) is a leading cause of preventable morbidity and mortality worldwide and is frequently complicated by psychiatric comorbidity, particularly major depressive disorder (MDD). Nearly 40% of individuals with AUD meet criteria for lifetime MDD, and co-occurring AUD and MDD is associated with greater disability, elevated relapse risk, and poorer outcomes than either disorder alone (58, 61). Despite this burden, interventions that simultaneously address alcohol use and depressive symptoms show limited and inconsistent effectiveness (57, 59, 63). A key contributor is the heterogeneity of AUD and its frequent co-occurrence with affective dysregulation, which complicates treatment matching and may obscure subgroup-specific mechanisms. These challenges have motivated calls for integrated, neurobiologically informed approaches that target shared and state-dependent mechanisms across disorders (65).

Neuromodulation, particularly transcranial magnetic stimulation (TMS), has been increasingly investigated as a potential intervention for substance use and mood disorders. In this review, “TMS” refers broadly to repetitive TMS (rTMS), including patterned protocols such as intermittent and continuous theta burst stimulation (iTBS, cTBS), as well as deep TMS and accelerated stimulation protocols. In AUD, rTMS studies have most commonly targeted the dorsolateral prefrontal cortex (DLPFC), given its role in executive control, decision-making, and craving regulation (1–3). While some trials report reductions in craving and drinking outcomes, findings remain heterogeneous, with modest and variable effects (4, 51). Most studies rely on region-centric targeting and do not incorporate neuroimaging to verify circuit engagement or mechanism (see Table 1). As summarized in Table 1, TMS studies in AUD are characterized by predominant DLPFC targeting, methodological heterogeneity, and mixed clinical outcomes despite evidence of neural engagement. Given the extensive and comparatively more standardized TMS literature in MDD [e.g., (5, 6)], we focus on AUD to highlight greater variability in targets and study design. These limitations raise questions about whether current paradigms adequately capture distributed circuit dysfunction in AUD, particularly in the context of comorbid depression. Recent consensus efforts emphasize the feasibility and clinical relevance of TMS in individuals with co-occurring substance use, but primarily address implementation and safety rather than mechanism (7, 8). Together, these findings underscore the need for mechanistically informed approaches to optimize target selection and engagement.

**Table 1:** Summary of transcranial magnetic stimulation (TMS) studies in alcohol use disorder (AUD), including stimulation targets, study design, imaging approaches, and behavioral outcomes.

**Study**	**N**	**Primary target**	**Stimulation type**	**Design**	**Type (Trial or Probe)**	**Neuroimaging modality**	**Outcomes**
Addolorato et al.(1)	14	Left DLPFC	rTMS	Randomized, sham-controlled	Trial	Task-based fMRI (activation)	Drinking; craving
Belgers et al.(42)	34	Right DLPFC	rTMS	Randomized, sham-controlled	Trial	None	Craving; drinking (long-term follow-up)
Ceccanti et al.(2)	18	mPFC	dTMS	Randomized, double-blind, sham-controlled	Trial	None	Craving; alcohol use; neuroendocrine markers
Del Felice et al. (43)	17	Left DLPFC	rTMS (high-frequency)	Single-blind, sham-controlled	Trial	EEG (resting-state spectral power)	Craving; inhibitory control; mood
Durazzo, Beauregard et al. (44)	44	Left DLPFC	iTBS	Randomized, sham-controlled	Trial	Structural MRI (VBM); MRS	Alcohol consumption
Durazzo, Kraybill et al. (45)	49	Left DLPFC	iTBS	Open-label	Trial	None	Abstinence
Girardi et al.(30)	20	Bilateral DLPFC	dTMS	Open-label	Trial	None	Craving; depressive symptoms
Hanlon et al. (46)	24	Left Frontal Pole	cTBS	Randomized, sham-controlled	Probe	TMS-evoked BOLD response (event-related fMRI)	Craving
Harel et al.(47)	46	mPFC + ACC	dTMS, H7 coil	Randomized, sham-controlled	Trial	Resting-state fMRI (functional connectivity)	Heavy drinking days; craving
Herremans et al.(33)	36	Right DLPFC	rTMS (high-frequency)	Single-blind, sham-controlled	Probe	None	Craving
Herremans et al. (3)	29	Right DLPFC	rTMS (high-frequency)	Single-blind, sham-controlled (crossover)	Probe	None	Craving
Herremans et al.(31)	26	Right DLPFC	rTMS (high-frequency; accelerated)	Sham-controlled + open-label	Trial	Task-based fMRI (cue-reactivity)	Cue-induced craving; general craving
Herremans et al.(48)	19	Right DLPFC	rTMS (high-frequency; accelerated)	Open-label feasibility	Trial	Task-based fMRI (cue-exposure)	Craving; relapse
Höppner et al. (34)	19	Left DLPFC	rTMS	Randomized, sham-controlled	Trial	None	Craving; mood
Hoven et al. (49)	80	Right DLPFC	rTMS (high-frequency)	Single-blind, randomized, sham-controlled	Trial	None	Abstinent days; craving; relapse; alcohol consumption
Jansen et al.(50)	38	Right DLPFC	rTMS	Randomized, sham-controlled	Probe	Resting-state fMRI (connectivity)	Alcohol-related problems; symptom severity
Jansen et al.(32)	39	Right DLPFC	rTMS (high-frequency)	Single-blind, sham-controlled	Probe	Task-based fMRI (emotion processing)	Craving; emotion processing
Kearney-Ramos et al.(37)	24	vmPFC	cTBS	Single-blind, sham-controlled (crossover)	Probe	Task-based fMRI (activation) + connectivity	Craving
McCalley et al. (51)	50	mPFC	cTBS	Randomized, double-blind, sham-controlled	Trial	Task-based fMRI (activation) + connectivity	Drinking outcomes; cue-reactivity
McNeill et al. (52)	20	Right DLPFC	cTBS	Within-subject, sham-controlled	Probe	None	Inhibitory control; alcohol consumption
Mishra et al. (53)	45	Right DLPFC	rTMS (high-frequency)	Single-blind, sham-controlled	Trial	None	Craving
Mishra et al. (54)	20	Right vs. Left DLPFC	rTMS (high-frequency)	Randomized, double-blind (active comparator)	Trial	None	Craving
Padula et al. (4)	6	dACC + dmPFC	dTMS, H7 coil	Open-label	Trial	Task-based fMRI (activation and connectivity)	Drinking
Perini et al. (35)	45	Insular cortex (bilateral)	dTMS	Randomized, double-blind, sham-controlled	Trial	Resting-state fMRI; task-based fMRI	Craving; alcohol use
Raikwar et al. (36)	60	Left DLPFC	rTMS (high-frequency)	Single-blind, randomized, sham-controlled	Trial	None	Craving
Wu et al. (55)	22	Right DLPFC	rTMS (high-frequency)	Open-label (exploratory)	Trial	Structural MRI (VBM)	Relapse; gray matter volume
Zhang et al.(56)	48	Left DLPFC	rTMS (high-frequency)	Randomized, double-blind, sham-controlled	Trial	None	Craving; alcohol use

Studies are organized by publication year and summarize sample characteristics, stimulation parameters, cortical targets, study design, neuroimaging modality, and outcomes. The majority of studies targeted the dorsolateral prefrontal cortex (DLPFC) using high-frequency repetitive TMS protocols, with substantial heterogeneity in stimulation parameters, targeting strategies, and outcome measures. Only a minority of studies incorporated neuroimaging to assess target engagement or mechanistic effects. Overall, findings demonstrate mixed clinical outcomes despite evidence of neural modulation, underscoring ongoing uncertainty regarding optimal targets, stimulation parameters, and circuit-level mechanisms in AUD neuromodulation. N = total sample size; ACC = anterior cingulate cortex; cTBS = continuous theta burst stimulation; dACC = dorsal anterior cingulate cortex; DLPFC = dorsolateral prefrontal cortex; dmPFC = dorsomedial prefrontal cortex; dTMS = deep transcranial magnetic stimulation; EEG = electroencephalography; fMRI = functional magnetic resonance imaging; GMV = gray matter volume; iTBS = intermittent theta burst stimulation; mPFC = medial prefrontal cortex; MRS = magnetic resonance spectroscopy; VBM = voxel-based morphometry; vmPFC = ventromedial prefrontal cortex. “Trial” denotes studies evaluating clinical or behavioral outcomes following repeated stimulation, whereas “Probe” denotes studies designed to assess acute or mechanistic effects. “Connectivity” (i.e., functional connectivity) refers to correlation-based (non-directional) measures unless otherwise specified.

Neuroimaging and translational research indicate that AUD and MDD share disruptions across large-scale networks involved in salience processing, attentional control, and cognitive flexibility [e.g., (9, 10)]. Neurobiological models conceptualize addiction as a disorder of interacting circuits governing incentive salience, negative emotionality, and executive function, with negative affect driving relapse via negative reinforcement (10, 11). Within this framework, the ventral attention network (VAN), closely related to salience network models, integrates interoceptive, emotional, and environmental signals to guide adaptive behavior (12, 13). Dysfunction within this network has been linked to impaired reorienting of attention away from negatively valenced internal states and salient external cues toward goal-directed behavior, a process central to relapse vulnerability in AUD and symptom persistence in MDD (9, 10, 14).

Recent large-scale studies further support the VAN as a shared neural substrate across AUD and MDD. Here, “VAN dysconnectivity” refers to altered functional connectivity within and between VAN nodes, typically assessed using resting-state (and, where noted, task-based) fMRI; unless otherwise specified, this refers to correlation-based (non-directional) connectivity. VAN dysconnectivity mediates associations between alcohol use severity and depressive symptom burden, and individuals with co-occurring AUD and MDD show more pronounced disruptions than those with either disorder alone (15, 16). These findings suggest that the VAN may represent a compelling target for transdiagnostic neuromodulation.

Against this backdrop, there is growing interest in moving beyond symptom-driven, region-specific stimulation toward paradigms that probe and modulate distributed networks. This Mini Review synthesizes the rTMS literature in AUD with co-occurring MDD and proposes a network-based framework centered on the VAN. We conceptualize TMS as a mechanistic probe of network responsivity and plasticity, emphasizing target engagement, state dependence, and individualized circuit dynamics (17, 18, 60). We highlight key gaps in current paradigms and discuss how integrating neuroimaging with neuromodulation may advance mechanistic understanding and support the development of more precise, biologically grounded approaches for this clinically complex population.

## The ventral attention / salience network as a transdiagnostic target

The ventral attention network (VAN) is a large-scale brain network involved in detecting behaviorally relevant stimuli and reorienting attention toward salient internal and external cues. In psychiatric neuroscience, closely related circuitry is often described as the salience network, reflecting overlapping roles in salience detection and network switching. While these frameworks overlap, VAN terminology, rooted in attention neuroscience, emphasizes stimulus-driven attentional reorienting and the central role of the temporoparietal junction (TPJ) alongside inferior frontal regions (12, 13). In contrast, salience network models more often center on the anterior insula and anterior cingulate cortex (ACC), with greater emphasis on affective and interoceptive processing (19, 20).

**Figure 1:**
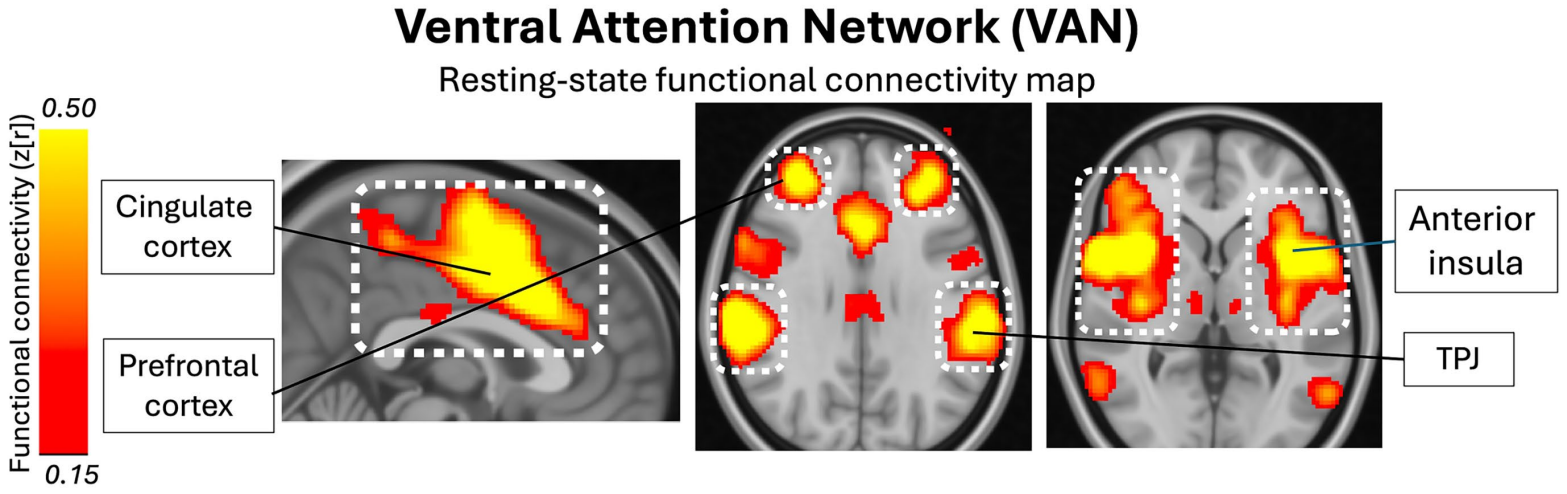
Ventral attention network (VAN) connectivity. Resting-state functional connectivity map illustrating core cortical VAN regions, including the temporoparietal junction (TPJ), anterior insula, anterior cingulate cortex (ACC), and lateral prefrontal cortex. The color scale reflects functional connectivity strength (Fisher *z*-transformed correlation coefficients). The VAN exhibits functional coupling with additional subcortical and associative regions not shown.

We define key VAN nodes as cortical regions showing intrinsic resting-state functional connectivity with the anterior insula and dorsal ACC, including lateral prefrontal regions (posterior inferior frontal gyrus and inferior frontal junction) and a parietal node near the TPJ (Figure 1B). These nodes are functionally coupled with subcortical and associative regions, including thalamic, cingulate, orbitofrontal, medial prefrontal, and cerebellar systems (12, 20–22). Here, we adopt the VAN framework to emphasize attentional reorienting and the TPJ as a neuromodulation-accessible network node, while acknowledging its close relationship to salience network models.

Disruptions in VAN circuitry are implicated in both AUD and MDD. In AUD, altered VAN connectivity is associated with impulsivity, impaired inhibitory control and working memory, and inefficient decision-making (23–25, 64). Structural alterations in key nodes, particularly the anterior insula and ACC, are linked to relapse risk, potentially reflecting reduced capacity to maintain control over urge-driven behavior during abstinence (26). These findings align with models of addiction in which compulsive alcohol use reflects dysregulated interactions among reward salience, negative affect, and executive control, with negative affect driving relapse [(10, 11); Figure 1].

In MDD, VAN dysfunction is associated with altered salience-related connectivity and disrupted coordination with default mode and executive control networks, contributing to rumination, affective bias, and reduced cognitive flexibility (9, 14). These findings reflect distributed network-level disturbances rather than isolated regional effects, highlighting shared mechanisms across AUD and MDD.

Recent large-scale analyses further support the VAN as a transdiagnostic system linking alcohol use and depressive symptoms. VAN dysconnectivity mediates associations between alcohol use severity and depressive symptom burden, and individuals with co-occurring AUD and MDD show more widespread disruptions than those with either disorder alone (15, 16). These findings suggest additive or synergistic effects of comorbidity on salience processing and attentional control.

The rationale for VAN-targeted neuromodulation rests on its role in stimulus-driven attentional reorienting, linking salience detection with downstream cognitive control. In AUD with co-occurring MDD, relapse vulnerability is often characterized by difficulty disengaging attention from negatively valenced internal states and salient external cues, resulting in persistent maladaptive patterns. While other approaches target executive control or affective processing more directly (e.g., DLPFC or medial prefrontal cortex), the VAN occupies an intermediate position that governs switching between internal and external attention and supports adaptive allocation of cognitive resources. Targeting this network, particularly via the TPJ, offers a mechanistically grounded approach to modulating processes that link salience attribution, attention, and behavioral flexibility.

**Figure 2:**
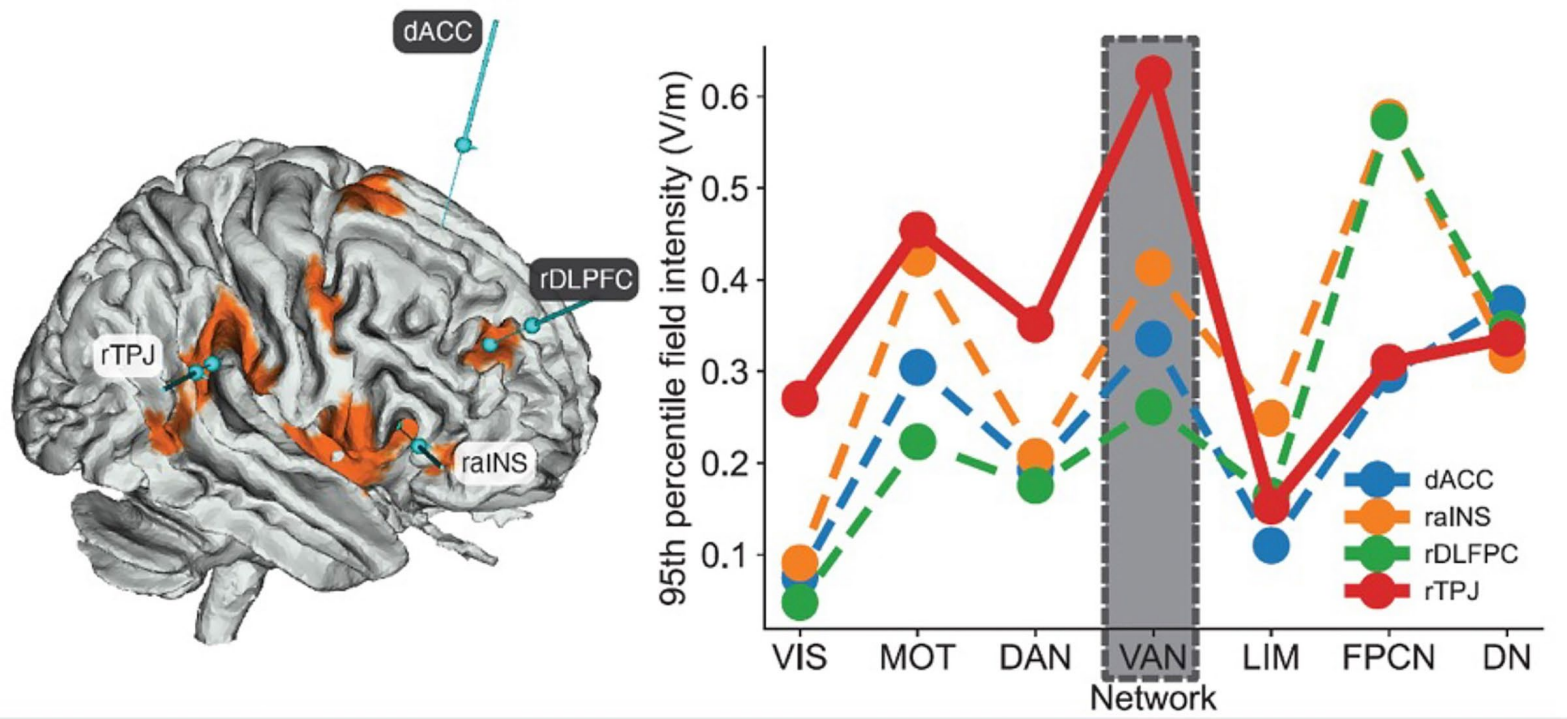
Optimization of target selection for ventral attention network (VAN) stimulation. Electric-field modeling (simulated electric field distribution) illustrates preferential engagement of cortical VAN circuitry during transcranial magnetic stimulation of the right temporoparietal junction (TPJ). The TPJ provides a neuromodulation-accessible cortical entry point for probing stimulus-driven attentional reorienting while minimizing activation of adjacent networks. VAN boundaries are shown for reference.

Within the VAN, the TPJ is a particularly accessible node for mechanistic investigation. It plays a central role in attentional reorienting, contextual updating, and integration of perceptual and affective information, linking salience detection to downstream attentional and executive systems (12, 13). Unlike deeper nodes such as the anterior insula or ACC, the TPJ is superficially located and accessible to focal TMS. Electric-field modeling (Figure 2) suggests that TPJ stimulation may preferentially engage VAN circuitry while minimizing spread to adjacent networks (27).

Prior work has proposed the salience network broadly as a neuromodulation target in AUD (18). Here, we emphasize the TPJ as a theoretically grounded and pragmatically accessible VAN node for probing network responsivity and plasticity. This focus follows from attention neuroscience models that position the TPJ as a core hub for attentional reorienting and contextual updating. Targeting the TPJ enables interrogation of how perturbations of attentional processes influence broader network dynamics during clinically relevant states, such as early abstinence with heightened negative affect.

Taken together, evidence from attention neuroscience, addiction research, and mood disorder studies positions the VAN, and the TPJ in particular, as a promising locus for network-based investigation. By coordinating interactions among salience, attention, and control networks, the VAN may exert a disproportionate influence on behavioral flexibility during periods of stress and relapse vulnerability. This perspective provides a foundation for neuromodulation paradigms aimed at elucidating mechanisms of recovery in individuals with AUD and co-occurring MDD.

## Network-based neuromodulation: current paradigms, gaps, and controversies

These patterns highlight a key limitation of existing approaches: current TMS paradigms in AUD largely focus on discrete cortical targets without accounting for distributed network dynamics. TMS has emerged as a potential adjunctive approach for AUD, motivated by evidence that maladaptive cortical–subcortical circuitry contributes to craving, impaired inhibitory control, and relapse vulnerability (10, 11, 28). Most neuromodulation strategies in AUD adapt paradigms developed for major depressive disorder, typically targeting the dorsolateral prefrontal cortex (DLPFC) within a “top-down control” framework (29, 62).

Consistent with this approach, most rTMS studies in AUD apply high-frequency stimulation to the left or right DLPFC over multiple sessions (Table 1). Some trials report reductions in craving and drinking outcomes, but findings remain heterogeneous, with variable effect sizes and durability (1, 30–32). Null or inconclusive results are also common, particularly in smaller studies or those with limited dosing and follow-up (33–36).

Methodological heterogeneity further contributes to this variability. Studies differ in stimulation frequency, intensity, coil type, number of sessions, and targeting strategy, often relying on coarse anatomical localization rather than individualized or functionally guided approaches (6). Most trials also assess behavioral outcomes without concurrent neuroimaging to verify circuit engagement, limiting mechanistic interpretability and cross-study comparability (Table 1).

A related limitation is that DLPFC stimulation primarily targets executive control processes and does not directly address salience attribution, affective bias, or integration of interoceptive and emotional signals, processes central to relapse vulnerability during early abstinence (11, 12). This regional focus may be particularly limiting in individuals with co-occurring AUD and MDD, where negative affect and attentional dysregulation play key roles in maintaining alcohol use (9, 14, 15).

Efforts to expand targets beyond the DLPFC, including medial prefrontal and insular regions, reflect recognition of these limitations (18, 35, 37). However, these approaches remain relatively sparse, often involve deeper or less accessible targets, and are rarely evaluated within a network-level framework. Few studies also account for patient state at the time of stimulation, despite evidence that neuromodulatory effects are state-dependent and vary with affective and cognitive context (17, 38).

Recent work has explored alternative strategies, including medial prefrontal and anterior cingulate cortex stimulation using deep TMS coil designs (39), as well as connectivity-guided and accelerated protocols (40, 41). These approaches aim to engage circuits involved in affective processing and network dysfunction but remain focused on modulating specific nodes. In contrast, a VAN-centered framework emphasizes stimulus-driven attentional reorienting as a dynamic process linking salience detection with downstream cognitive control, offering a complementary network-level perspective on target engagement.

Collectively, this literature highlights a central controversy: whether neuromodulation in AUD should prioritize repeated stimulation of canonical prefrontal targets to achieve clinical effects, or shift toward mechanistic, network-level approaches that better capture distributed circuitry. The latter emphasizes probing network responsivity and plasticity as candidate biomarkers, rather than focusing solely on symptom reduction, and may be particularly informative in populations with severe comorbidity.

Together, these considerations support a shift from region-centric, symptom-driven stimulation toward approaches grounded in large-scale network organization and circuit dynamics. In contrast to salience network–focused frameworks, we adopt a VAN framework that emphasizes stimulus-driven attentional reorienting and identifies the TPJ as a neuromodulation-accessible network node. Reframing TMS as a probe of VAN responsivity and plasticity offers a principled approach to addressing heterogeneity, state dependence, and distributed circuit dysfunction in AUD with co-occurring MDD.

## Discussion and future directions

This Mini Review synthesizes evidence supporting a network-based perspective on neuromodulation in AUD, with emphasis on the ventral attention network (VAN) and its relevance to co-occurring MDD. While prior work has highlighted the salience network as a promising neuromodulation target in AUD (18), this review extends that literature by shifting the focus from target identification toward probing network responsivity and plasticity. This reflects a broader reframing of TMS as a tool for interrogating circuit dynamics rather than solely reducing symptoms.

A central contribution is the emphasis on the temporoparietal junction (TPJ) as a theoretically grounded and pragmatically accessible VAN node. Drawing from attention neuroscience, the TPJ plays a key role in stimulus-driven attentional reorienting and contextual updating (12, 13), processes that are particularly vulnerable during early abstinence and heightened negative affect. Unlike deeper VAN nodes such as the anterior insula or anterior cingulate cortex, the TPJ’s superficial location enables focal stimulation and integration with neuroimaging-guided targeting. This focus complements prior salience network–oriented work by providing a specific cortical entry point for interrogating VAN dynamics.

The network responsivity framework addresses several limitations of current neuromodulation paradigms in AUD. Region-centric approaches, most commonly targeting the dorsolateral prefrontal cortex, have yielded mixed outcomes and offer limited insight into distributed circuitry underlying relapse vulnerability. This has contributed to debate over whether inconsistent findings reflect suboptimal targets, insufficient network engagement, or heterogeneity in underlying mechanisms. Assessing how large-scale networks reorganize following perturbation provides a mechanistic lens for understanding individual differences in plasticity and recovery potential. This perspective aligns with evidence that neuromodulatory effects are state-dependent, varying with baseline activation, affective context, and cognitive demands (17, 38), and is particularly relevant for individuals with AUD and co-occurring MDD.

Future research should prioritize several directions. First, studies should examine network-level responses to neuromodulation across clinically meaningful states, including early abstinence, stress, and negative affect. Second, integrating neuroimaging with stimulation protocols will be essential to verify target engagement and distinguish circuit-specific from nonspecific effects. Third, longitudinal work is needed to determine whether measures of network responsivity and plasticity predict outcomes such as relapse, remission, and treatment engagement. The VAN–TPJ framework does not imply exclusivity of this network, nor does it preclude contributions from circuits involved in habit learning, reward valuation, or stress responsivity.

More broadly, a network-based neuromodulation paradigm may inform precision approaches to addiction treatment. Rather than assuming uniform benefit from repeated stimulation of a canonical cortical region, interventions could be tailored to individual patterns of network dysfunction and plastic response. This perspective aligns with models of large-scale brain organization emphasizing dynamic interactions among attention, salience, and executive control systems (19). While such applications remain speculative, this framework provides a foundation for systematic, mechanistically informed investigation.

In summary, reframing neuromodulation as a probe of network dynamics highlights new opportunities for advancing both scientific understanding and clinical translation of brain stimulation in AUD and co-occurring MDD. By integrating insights from attention neuroscience, addiction theory, and systems-level neuroimaging, this perspective underscores the potential value of the VAN, particularly the TPJ, as a locus for investigating mechanisms of recovery and relapse vulnerability. Continued work may inform more targeted, biologically grounded strategies for supporting recovery in complex clinical populations.
